# Miscellaneous

**Published:** 1889-04

**Authors:** 


### MISCELLANEOUS.

Hypnotism.—Two gentlemen interested in psychological studies, Mr. W. A. Croffut, executive officer of the Geological Survey, and Gov. N. J. Coleman, Commissioner of Agriculture, give occasional *soirees hypnotiques* in Washington, at which they hypnotize numbers of "sensitives." During some recent experiments by Mr. Croffut, two young ladies, temporary victims of the hypnotic hallucination, were taken into an imaginary picture gallery and there left, while the operator turned his attention to a young man who was engaged in the dangerous pastime of catching crocodiles. On returning to the ladies Mr. Croffut found that he could not make them cognizant of his presence. They did not appear to see him, or hear his voice, and when he stood directly in front of them they took no notice of him whatever. It was a new and somewhat alarming experience, and a quarter of an hour passed before the hypnotizer re-established his domination and brought them back from the land of dreams.—*Science*.

A Wonderful Mirage.—There is reported to be a wonderful mirage in Glacier Bay, Alaska, reflected from the glassy surface of the Pacific glacier. Just after the change of the moon, in June, soon after sunset, and while the moon is climbing above the sky, a city appears above the glacier. It is so distinct that a photograph is said to have been made this season by a resident of Juneau, who learned of the mirage from the Indians, and has seen it appear and disappear for four years.

To Cement Leather to Iron.—Paint the iron with white lead and lamp black, when dry it is covered with a cement made by dissolving the best glue in vinegar and adding about one-third of turpentine. Apply the cement warm.

Paste for Labels.—Mix a few drops of butter of antimony with mucilage of acacia.

To Color Ivory.—Immerse the articles ten to twenty minutes in dilute nitric acid (1 in 32), then the same length of time in solution of stannous chloride (1 in 200), in a mixture of solution of carmine (1 in 200) and ammonia (q. s.) When dry and cold, polish with boiled linseed oil. For blue, use Prussian blue instead of carmine, for yellow, either turmeric or safflower. For brown use a mordant of acetate of alumina and acetate of iron, and dye with madder or fustic. Analine dyes may also be used.

Allen's Excelsior Axle Grease.—

| | |
|---|---|
| Castor Oil, | 1 quart. |
| Linseed Oil, | 1 " |
| Tallow, | 2 lbs. |
| Resin, | 2 " |
| Beeswax, | 1 lb. |

If too hard, add a little neatsfoot oil, if too soft add tallow.

Deafness of Old Age.—Sapolina, of Milan, describes a method of his, which he states he has successfully employed in sixty-two cases of deafness of old age. It consists in mopping the membrana tympani with a weak oleaginous solution of phosphorous. He claims that this treatment diminishes the opacity of the membrane, increases the circulation and improves the hearing.

Cure for Dandruff.—Mr. John L. Davis, in the *Journal of Pharmacy*, asserts (having fully tested it in his own case), that a preparation of one ounce of sulphur and one quart of water, repeatedly agitated during intervals of a few hours, and the head saturated every morning with the clear liquid, will, in a few weeks, remove every trace of dandruff from the scalp, and the hair will become soft and glossy. He says: "I do not pretend to explain the *modus operandi* of the treatment, for it is well known that sublimed sulphur is almost or wholly insoluble, and the liquid used was destitute of taste, color, or smell. The effect speaks for itself."

A Simple Medicine.—A prominent physician was seen buying a barrel of onions, and being guyed about his purchase, said: "I always have boiled onions for dinner for the benefit of my children. No worms, no scarlatina, no diphtheria, where children eat plentifully of onions every day."

It is not five years since the clearest practical demonstrations of the economy of burning water hydrogen were the laughing stock of book philosophy, and probably they are so still. Nevertheless, this element has forced its way over the distrust sown by impracticable theorists, and by one process or another, worked with oil or coal, is giving profits to business in a great variety of situations.

